# The Effect of Implementation Intentions on Prospective Memory Commission Errors under Different Cognitive Loads

**DOI:** 10.3390/bs13070602

**Published:** 2023-07-19

**Authors:** Yunfei Guo, Jiaqun Gan, Yi Ding, Yongxin Li

**Affiliations:** Institute of Psychology and Behavior, Henan University, Kaifeng 475000, China; gyfhenu@126.com (Y.G.);

**Keywords:** prospective memory, implementation intention, commission error, encoding method, cognitive load

## Abstract

Prospective memory (PM) refers to the ability to remember to perform a planned event or activity at a specific time or situation in the future. Implementation intentions can promote a connection between PM cues and intended actions, thus improving an individual’s PM performance. However, this simple and effective encoding strategy may also have negative effects. For example, an implementation intention may result in PM commission errors that occur when an individual makes a false PM response to repeated PM cues that are no longer relevant as the PM task has been completed. Existing studies have explored the effect of implementation intentions on PM commission errors under low cognitive load. However, the role of implementation intentions in promoting linkages between PM cues and actions tends to disintegrate under high cognitive loads. Therefore, this study aimed to explore the effect of implementation intentions on PM commission errors under different cognitive load conditions. In this study, 58 college students participated in a mixed experimental design of 2 (encoding methods: implementation intention, standard) × 2 (cognitive load conditions: low, high). The results showed that implementation intentions promoted PM commission errors under the low-cognitive-load condition only, and there was no difference in the performance of ongoing tasks between the implementation intention encoding and the standard encoding conditions. However, individuals in the implementation intention condition reacted more slowly when encountering previous PM cues. The results suggest that the effect of implementation intentions on PM commission errors relies upon automated processing as a whole. However, individuals in the implementation intention condition required more attentional resources to suppress the no-longer-relevant intended actions when previous PM cues appeared, supporting the dual-mechanism theory.

## 1. Introduction

Prospective memory (PM) refers to the ability to remember to execute a planned event or activity at a specific time or situation in the future [1]. An example would be to remember to buy bread when passing a convenience store. However, an individual will sometimes repeat the previous PM behavior when they encounter PM cues (e.g., buying bread twice) that are no longer relevant, which is called a PM commission error [2]. Several theories attempt to explain the processes underlying commission errors. The automated processing view attributes the occurrence of commission errors to the strong connection between PM cues and the behavior, which promotes the automated execution of a PM response. When the previous PM task is cancelled, individuals are likely to spontaneously extract the previous PM intention when encountering the previous PM cue. The stronger the connection between the PM cue and intentional behavior, the greater the probability of individuals spontaneously extracting the previous PM intention. It is a process that does not require the consumption of attentional resources [3,4]. By comparison, the monitoring view contends that individuals continue to monitor previous PM cues after completing the PM task, and commission errors arise from devoting sustained attention to monitoring cues. The more they monitor the original PM cues, the greater the probability of commission errors [2]. The inhibition view suggests that individuals continue to inhibit the previous PM intention after completing the PM task, and a commission error will occur if attentional resources are insufficient to inhibit the behavior of the previous PM tasks. The dual mechanism view holds that the strong connection formed between PM intention and behavior is the basis for the occurrence of commission errors in prospective memory. Not paying enough attention to suppressing the previous PM intention is the direct cause of commission errors [5].

To date, research into the factors that influence PM commission errors has mainly focused on the retrieval process associated with the PM intention, and few studies have focused on the effects of the encoding process on PM commission errors. Implementation intention is an encoding strategy that closely links the expected situation with the intended actions, which may have an impact on PM commission errors. This strategy involves encoding tasks in the form of “if-then” (if situation X appears, then I will perform action Y) [6]. Studies have shown that implementation intention is an effective PM encoding strategy, which can form a strong cue-behavior encoding association, thereby improving PM performance [6]. The implementation intention can be viewed as a double-edged sword, and while it is an effective encoding strategy that promotes PM performance, it may also increase commission errors after the PM task is completed. Commission errors may have adverse consequences in certain situations, for example, double dosing of one’s medication might result in harm. Therefore, the investigation of the potential mechanisms by which implementation intentions promote PM commission errors is of great practical importance, and an improved understanding of the factors that influence commission errors may suggest ways to reduce their occurrence.

Although implementation intention encoding significantly improves PM performance, it also increases the repeated execution of completed intentions that are no longer relevant, thus producing the risk of commission errors. Based on the automated processing view, implementation intention can promote the automatic connection between PM cues and behaviors. This strong connection may remain in a stable activation state after the PM task is completed, which promotes the automated processing of completed intentions and results in commission errors. As far as we know, only two studies have investigated the effects of implementation intentions on PM commission errors. Meiser and Rummel compared the differences in PM commission errors between an implementation intention condition, a mixed condition (implementation intention + practice), and a control condition in young adults. The results showed that individuals in the implementation intention and mixed conditions had a higher probability of commission errors compared with the control condition. The authors believed that implementation intention encoding enhanced the automated processing of PM responses, and the occurrence of PM commission errors also resulted from automated processing [7]. Another study also investigated differences in the effect of implementation intention encoding on PM commission errors in older and young adults. They found that implementation intention encoding significantly increased commission errors by both older and young adults compared to standard encoding [8].

Although existing studies have found that implementation intention can promote the occurrence of PM commission errors, they were all carried out under the condition of low cognitive load, requiring fewer attentional resources (the ongoing tasks used were simple lexical decision tasks). For instance, Mesier and Rummel’s ongoing task accuracy was more than 0.95, indicating a possible ceiling effect [7], while Bugg et al. did not report the performance of ongoing tasks, but the ongoing tasks were also simple lexical decision tasks [8]. Although a large number of studies found that implementation intention could promote the connection between PM cues and intentional behavior, the promoting effect of implementation intention was easily influenced by attention load. Under the high-attention-load condition, the connection strengthened by implementation intention was prone to collapse [9]. Previous studies only explored the effect of implementation intention on PM commission errors under the low-attention-load condition, and we focused on the effect of implementation intention encoding on PM commission errors under different cognitive loads.

This study investigated the effect of implementation intentions on PM commission errors under different cognitive load conditions. The attention to the above issue is not only beneficial for clarifying the boundary conditions under which implementation intention affects PM commission errors, but also for further verifying the existing theoretical viewpoints. If implementation intention can increase the commission errors, it indicates that the occurrence of PM commission errors is related to a stronger connection between previous prospective memory intention and behavior, which can validate the viewpoint of automated processing. In addition, high attention load will reduce the available attention resources. The viewpoint of monitoring predicts that the lack of attention can lead to insufficient attention to the previous PM cues, which will reduce commission errors. The inhibitory viewpoint predicts that a lack of attention can lead to insufficient inhibition toward the previous PM intention, which will increase commission errors. Therefore, exploring the changes in commission errors under different attention loads can help distinguish between the inhibitory viewpoint and monitoring viewpoint. If we simultaneously discover that individuals in both the implementation intention encoding and high attention load conditions have a promoting effect on commission errors, the results will confirm the dual mechanism viewpoint [10].

## 2. Method

### 2.1. Participants and Design

Fifty-eight college students from Henan University participated in the experiment, of whom 50% were male (*n* = 29). Participants were aged from 18 to 26 years, with an average age of 20.56 years, and were randomly assigned to one of the two encoding conditions. All participants had normal or corrected vision and never participated in a similar experiment. The studies involving human participants were reviewed and approved by the Institutional Review Board of Henan Provincial Key Laboratory of Psychology and Behavior. Participants were required to sign an informed consent form before the experiment commenced and received a small reward (15 yuan) when the experiment was finished.

A mixed design of 2 (encoding condition: implementation intention, standard) × 2 (cognitive load: low, high) was adopted. The encoding condition was a between-subject variable, while cognitive load was a within-subject variable.

### 2.2. Apparatus and Stimuli

The experimental materials were 24 English capital letters (excluding the letters F and J that were reserved for responses), displayed in black font on a white background. The PM cues were the letters G and Q, and the ongoing task stimuli were randomly selected from the remaining 22 letters. The experiment used E-prime 2.0 to compile the program and present the experimental instructions, stimulus items, and collect data on a computer. Participants were tested individually in a sound-attenuated booth and performed the ongoing task and PM task response by pressing certain keys on the keyboard.

### 2.3. Procedure

The formal experimental procedure was divided into a PM active phase and a PM finished phase [9]. In the PM active phase, two tasks were performed at the same time: the ongoing task and the PM task. By comparison, only the ongoing task was performed in the PM finished phase. The ongoing task employed the n-back (*n* = 1,2) paradigm to manipulate cognitive load. Under the low-cognitive-load condition, participants were only required to implement the 1-back task, while participants performed the 2-back task under the high-cognitive-load condition. In the 1-back task, participants were asked to compare whether the current letter on the screen was the same as the first letter that followed it. If they were the same, participants were asked to press the J key; otherwise, they were to press the F key. In the 2-back task, participants were required to compare the current letter with the second letter that followed it and respond in the same way as in the 1-back task. To balance any potential order effects, half of the participants undertook the 1-back part first, followed by the 2-back part, while the remaining participants completed the two tasks in the reverse order. The PM task was to press the space bar when the letter G or R appeared on the screen.

The PM instruction for the standard encoding condition was “when you see the letter G or R, you don’t need to perform the letter comparison task, just press the spacebar”. The PM instruction for the implementation intention encoding condition was “if you see the letter G or R, then you should immediately press the spacebar and there is no need to perform the letter comparison task” (using the “If then” encoding form). And after determining that the participant understood the instructions, they were asked to verbally repeat the PM task instruction three times loudly [11]. In addition, both conditions added a pen-and-paper test of the PM task after encoding: “If you see (), then press the () key” to further ensure that participants had formed an encoding connection.

In the experiment, the ongoing task instruction was presented first, and participants commenced the practice phase (50 ongoing task trials without PM task) after it was apparent that they correctly understood the instruction. The stimulus was initiated by first presenting the fixation point “+” (500 ms) in the center of the screen, followed by a capital letter (2000 ms, which disappeared immediately after the participant responded). After completing the practice phase, the PM instructions were displayed on the screen, and when participants understood what was required, they were told that the formal experiment would consist of two phases: the first required performing the PM task while the second did not.

The PM active phase included 80 ongoing task trials, in which 4 PM cue trials were inserted, R and G twice each. At least 10 ongoing task trials separated each two PM cues, which was followed by five minutes of complex verbal numerical calculations as a distraction task. After that, the PM finished phase began.

The PM finished phase comprised 150 ongoing task trials, of which 8 PM cue trials were inserted, R and G 4 times each. After the experiment, the participants were asked to recall the target words and response keys from the active phase to confirm that the participants had not forgotten the PM task content.

## 3. Results

The PM accuracy during the active phase was defined as the rate at which a participant correctly pressed the space bar when confronted with a PM cue, and the PM commission error rate was defined as the rate at which participants mistakenly pressed the space bar when encountering previous PM cues. A series of repeated-measures analysis of variance (ANOVA) were performed to assess whether there was any effect of the encoding condition (standard versus implementation intention), the cognitive load condition (high versus low), or any interaction between them on any outcome variable.

### 3.1. Performance of PM Task and Ongoing Task in the Active Phase

Results of the ANOVA showed that there was a significant main effect of cognitive load on PM accuracy, *F*(1, 56) = 43.79, *p* < 0.001, *η_p_*^2^ = 0.44, but the main effect of the encoding condition was non-significant, *p* > 0.05. There was also a significant interaction between cognitive load and the encoding condition, *F*(1, 56) = 4.54, *p* < 0.001, *η_p_*^2^ = 0.08. Further simple effects analysis found that the accuracy of implementation intention encoding in the low-cognitive-load condition was higher than that in the standard encoding condition, *p* < 0.05, while there was no difference between them in the high-cognitive-load condition, *p* > 0.05. In the standard encoding condition, the PM accuracy was lower in the low-cognitive-load condition than that in the high-cognitive-load condition, *p* < 0.05, but there was no difference between the two cognitive loads in the implementation intention encoding, *p* > 0.05.

The results also showed that there was a significant main effect of cognitive load on PM reaction time, *F*(1, 56) = 104.76, *p* < 0.001, *η_p_*^2^ = 0.65, with the speed under low cognitive load being faster than that under high cognitive load. A significant main effect of the encoding condition was also found, *F*(1, 56) = 5.50, *p* < 0.05, *η_p_*^2^ = 0.09, and the reaction time of implementation intention encoding was significantly faster than in the standard encoding condition. The interaction between cognitive load and the encoding condition was not significant, *p* > 0.05.

Similarly, there was a significant effect of cognitive load on the accuracy and reaction time of the ongoing task, *F*(1, 56) = 58.67, *p* < 0.001, *η_p_*^2^ = 0.51, *F*(1, 56) = 58.67, *p* < 0.001, *η_p_*^2^ = 0.51, with higher accuracy and faster response speed under the low-cognitive-load condition than under the high-load condition. No other results were significant, *ps* > 0.05 (see Table 1).

### 3.2. PM Commission Error Rate and Previous PM Cue Reaction Time in the Finished Phase

The results also showed that there was a significant main effect of cognitive load on the PM commission error rate, *F*(1, 56) = 4.27, *p* < 0.05, *η_p_*^2^ = 0.07, but the encoding condition did not show a significant main effect, *p* > 0.05. There was a significant interaction between cognitive load and the encoding condition, *F*(1, 56) = 4.59, *p* < 0.05, *η_p_*^2^ = 0.08. Further simple effects analysis revealed that the commission error rate of implementation intention encoding was significantly higher than that of standard encoding under low cognitive load, *p* < 0.05, while there was no difference between the two under high cognitive load, *p* > 0.05.

**Figure 1:**
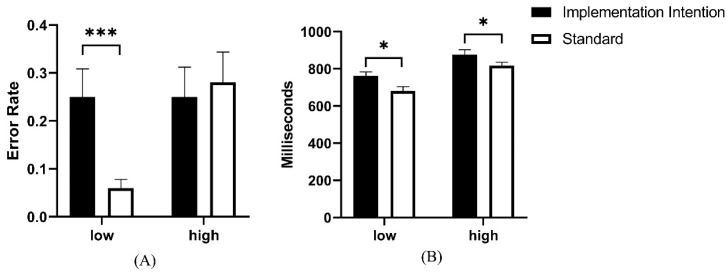
The PM commission error rate and the reaction time of previous PM cues in the finished phase. High represents high load and low represents low load. * *p* < 0.05, *** *p* < 0.001.

Finally, a significant main effect of cognitive load on the reaction time of the previous PM cue was found, *F*(1, 56) = 74.19, *p* < 0.001, *η_p_*^2^ = 0.57, and the response speed was faster under low cognitive load than that under high cognitive load. Likewise, the encoding condition significantly influenced reaction time, *F*(1, 56) = 5.16, *p* < 0.05, *η_p_*^2^ = 0.08, with the implementation intention encoding resulting in slower reaction speed than standard encoding. However, no significant interaction was found between encoding condition and cognitive load, *p* > 0.05 (see Figure 1 and Table 2).

### 3.3. Ongoing Task Accuracy and Reaction Time (Excluding Previous PM Cues) in the Finished Phase

The results indicated that the main effect of cognitive load on the ongoing task accuracy in the finished phase was significant, *F*(1, 56) = 148.99, *p* < 0.001, *η_p_*^2^ = 0.73, with higher accuracy in the low-cognitive-load condition compared to that in the high-load condition. However, no main effects of encoding condition and interaction between encoding condition and cognitive load were found, *ps* > 0.05.

Finally, the results also showed that the main effect of cognitive load on the ongoing task reaction time was significant, *F*(1, 56) = 90.99, *p* < 0.001, *η_p_*^2^ = 0.62, and the response speed was faster under the low-load condition than that under the high-cognitive-load condition. No other effects were found to be significant, *ps* > 0.05.

### 3.4. Comparison of Previous PM Cue Reaction Times versus Ongoing Task Reaction Times in the Finished Phase

The results showed that there was a significant main effect of cognitive load on reaction time, *F*(1, 112) = 164.70, *p* < 0.001, *η_p_*^2^ = 0.60, and reaction time was faster under the low-cognitive-load condition compared to that under the high-cognitive-load condition. The main effect of stimulus type was also found to be significant, *F*(1, 112) = 5.18, *p* < 0.05, *η_p_*^2^ = 0.04, and the response speed of previous PM cues was slower than that of the ongoing task. No other significant effects were revealed, *ps* > 0.05.

## 4. Discussion

The current study primarily explored the effect of implementation intentions on PM commission errors under the condition of high and low cognitive load by enhancing the strength of the connection between PM cues and behaviors. The results showed that during the active phase, an implementation intention improved PM performance under the low-cognitive-load condition only. This result verified our hypothesis and is consistent with the results of previous studies [11]. Our results from the active phase also support the view that an implementation intention can promote the connection between PM cues and behaviors, but the promoting effect is easily affected by cognitive load. Consistent with this, implementation intention encoding also resulted in a higher commission error rate than standard encoding under low-cognitive-load conditions only. This indicates the negative effect of an implementation intention on PM commission errors under the low cognitive load, also verifying viewpoint of automated processing. Although an implementation intention can promote the connection between PM cues and behaviors [6], the strength of this connection is unstable and easily disintegrates under the condition of high cognitive load. The effect is attenuated when it continues to the finished phase. Another possibility is that high attention load leads to a shortage of available attention resources for individuals, and the lack of attention used to suppress the previous prospective memory intention is the main reason for commission errors. Moreover, under the standard coding condition, the commission error rate has reached a relatively high level (*M* = 0.28), and its improvement space is already limited. Therefore, implementation intentions appear to have an impact on commission errors under low cognitive loads only.

The automated processing view asserts that commission errors occur because, there, a strong connection between PM cues and behaviors remains after the PM task has been executed. When an individual encounters a previous PM cue, it is easy to spontaneously retrieve the completed PM intention, resulting in a commission error [4]. This study strengthened the connection between PM cues and behaviors through an implementation intention and increased the individual’s PM commission error rates under low cognitive load, which partially supports the automated processing theory. At the same time, we found no difference between the implementation intention group and the control group in the accuracy and reaction times of the ongoing task. This finding suggests that the promotion of implementation intentions on PM commission errors was not accompanied by additional attention demands on the whole. This result also shows that the effect of the implementation intention on PM commission errors is a process of automated processing. It can be explained by the fact that although PM intention maintains a state of activation following completion of the PM task, the activation state is at a subthreshold level [4] and does not require significant attentional consumption.

However, we also found some evidence of controlled processing. For example, the response speed to previous PM cues was significantly slower in the implementation intention group than in the standard encoding group, suggesting that individuals took longer to process the task when they encountered previous PM cues. By comparison with the standard encoding group, the implementation intention encoding group did not allocate additional attention resources to the overall ongoing task, but only deployed additional attentional resources when encountering previous PM cues. Since the implementation intention strengthens the encoding connection between PM cues and behaviors [12], strong mental conflicts arise when individuals re-encounter PM cues that are no longer relevant during the execution of an ongoing task. Thus, they need more time and attention to think and make decisions. In addition, we found that individuals responded significantly more slowly to previous PM cues than to the ongoing task during the finished phase, which also indicated that more attention was required when encountering previous PM cues. Thus, the question arises: did individuals use this time to monitor previous PM cues or to inhibit completed PM intentions? We suspect the latter, because individuals do not know in advance when and where PM cues will appear, and if they want to monitor PM cues, they usually need to devote attentional resources to continuous cue monitoring throughout the experimental phase [13]. In contrast, the process of inhibition is different, insofar as individuals can suppress PM intentions throughout the process, as well as in the event of a mental conflict caused by repeated PM cues [14]. Based on this analysis, it appears that the implementation intention group may need more attentional resources to suppress the completed PM intention. In terms of PM commission error rates, we found that the commission error rates in the high-cognitive-load condition were higher than in the low-cognitive-load condition. Under high cognitive load, individuals had fewer attentional resources available and a higher rate of commission errors resulted, indicating that fewer attentional resources triggered more commission errors, which validated the viewpoint of inhibition. This finding is consistent with the inhibition argument that has also been validated in other studies. For example, some studies have found that older adults with an intact automated processing ability but poor inhibition ability are prone to commit more commission errors [15], and it follows that inhibition ability should also play a role in the occurrence of commission errors.

Combining the above analyses, we found evidence of both automated and inhibited processing, suggesting that both processes may explain the results of this study. This is consistent with the idea proposed by Scullin et al. [16], who argued that commission errors may result from a combination of the spontaneous retrieval of the completed PM intentions and the failure of individuals to inhibit the intention. A prerequisite for a commission error is a strong connection between PM cues and behaviors, prompting PM responses to be in a state of spontaneous retrieval. When individuals have insufficient resources for inhibition, they commit commission errors, and this view is termed the dual-mechanism account. Other studies have also found evidence for both automated and controlled processing [8], further supporting the dual-mechanism hypothesis.

The present study explored the effect of implementation intention on PM commission errors under different cognitive load conditions. Although there are some meaningful findings, there were some limitations to our study. First, participants were informed in advance of all the requirements of the experimental procedure in this study, which simulates a real-life PM task that requires advanced planning. However, there was another situation in which individuals were told that they no longer need to perform the completed PM task after completing the PM task in the active phase. In that case, individuals strongly inhibited the completed PM intention, leading them to make few or no commission errors [8]. Although implementation intention can promote the spontaneous retrieval of a completed PM intention, it is difficult for an implementation intention to facilitate commission errors when individuals have a high level of inhibition. Second, as in other studies, our study adopted the ongoing task performance to indirectly reflect changes in attention, but the effectiveness of this indicator was poor. Compared with a simple key response, eye tracking technology can directly reflect the object, frequency, and degree of individual attention [17], which can more effectively and comprehensively reflect changes in attention. It is suggested that future research should employ more advanced research techniques to further explore the impact of implementation intentions on PM commission errors. Third, the ongoing tasks used in this study are relatively simple, which is significantly different from the complex and diverse real-life situations. Especially, the high-attention-load condition we set did not fully occupy the individual’s attention resources (with an accuracy rate between 0.77 and 0.80), resulting in an ineffective manipulation of attention load. Therefore, future research should consider the ecological validity of tasks and the effectiveness of variable manipulation. Finally, the essence of implementation intention is to play a promoting role by strengthening the connection between prospective memory cues and intentional behavior. To further demonstrate the advantages of implementation intention encoding, we should present the instruction with the “if-then” form more intuitively and separately, rather than simply describing it in a lengthy and unfocused manner.

## 5. Conclusions

This study focused on the effects of implementation intention and cognitive load on PM commission errors. It was found that an implementation intention promoted PM commission errors under the low-cognitive-load condition only, and this promotion effect required no additional attentional resource consumption overall. However, individuals in the implementation intention encoding group expended more attention to inhibit completed PM intentions when previous PM cues were encountered. The results support the dual-mechanism theory. The current research not only provides a deeper understanding of the impact of implementation intentions on PM commission errors, but also further tested existing theories of PM commission errors, with theoretical significance.

## Figures and Tables

**Table 1:** The performance of prospective memory task and ongoing task in the active phase.

		Prospective Memory		Ongoing Task	
		ACC	RT (ms)	ACC	RT (ms)
Standard	Low	0.73 (0.19)	769 (152)	0.85 (0.06)	711 (128)
	High	0.56 (0.32)	952 (149)	0.75 (0.09)	875 (185)
II	Low	0.86 (0.20)	694 (104)	0.87 (0.05)	696 (107)
	High	0.53 (0.24)	892 (110)	0.76 (0.09)	839 (142)

II = implementation intention; Low = low load; High = high load.

**Table 2:** The performance of ongoing task under difference cognitive loads in the finished phase.

		Previous PM Cues		Ongoing Task	
		Error Rate	RT (ms)	ACC	RT (ms)
Standard	Low	0.06 (0.10)	681 (131)	0.90 (0.06)	641 (120)
	High	0.28 (0.35)	817 (106)	0.80 (0.04)	834 (104)
II	Low	0.25 (0.32)	761 (124)	0.90 (0.05)	663 (133)
	High	0.25 (0.34)	876 (151)	0.77 (0.09)	814 (130)

II = implementation intention; Low = low load; High = high load.

## Data Availability

The data that support the findings of this study are available from the corresponding author upon request.
